# Effectiveness of the ‘Who’s Challenging Who’ support staff training intervention to improve attitudes and empathy towards adults with intellectual disability and challenging behaviours: study protocol for a cluster randomised controlled trial

**DOI:** 10.1186/s13063-017-2175-1

**Published:** 2017-10-05

**Authors:** Elizabeth Randell, Richard P. Hastings, Rachel McNamara, Roseanna Knight, David Gillespie, Zachary Taylor

**Affiliations:** 1 Centre for Trials Research, Cardiff University, Cardiff, UK; 2 Centre for Educational Development, Appraisal, and Research (CEDAR) University of Warwick, Coventry, UK; 3 Royal Mencap Society, Unit 7 Sundon Business Park, Dencora Way, Luton, UK

**Keywords:** Attitudes, Challenging behaviour, Intellectual disability, Co-trainers, Contact hypothesis, Empathy, Staff

## Abstract

**Background:**

Findings suggest approximately one in six people with intellectual disability engage in ‘challenging behaviours’, which include aggression towards others/property and self-injurious actions. In residential settings, actions of staff members can make challenging behaviours more likely to occur, or make these behaviours worse. In particular, negative attitudes from members of staff and lack of understanding about the reasons for challenging behaviour are contributory factors. ‘Who’s Challenging Who?’ (WCW) training is designed to emphasise the role of staff in residential settings as a challenge also to people with intellectual disability. The course is delivered jointly by a trainer with intellectual disability who has been labelled as having challenging behaviour, along with a trainer without intellectual disability.

**Methods:**

This is a cluster randomised two-arm trial of WCW training versus a waiting list control. Overall, 118 residential settings will be recruited and randomised on a 1:1 ratio. Within each setting, two members of staff will be invited to take part in the trial. Participants will complete assessments at baseline and at 6 and 20 weeks. WCW is a half day initial training course with some follow-on coaching to ensure implementation. The primary outcome is changes in staff empathy towards people with challenging behaviour. Secondary outcomes at the staff level include confidence, attitudes and work-related well-being. Secondary outcomes at the residential setting level include recorded incidents of aggressive challenging behaviour, and use of any restrictive practices.

**Discussion:**

If the results of the cluster randomised trial are positive, we will disseminate the findings widely and make all training manuals and materials freely available for anyone in intellectual disability services (and beyond) to use. Our training approach may have wider implications in other areas of social care. It may also provide a generally applicable model for how to train people with intellectual disability to act as co-trainers in intellectual disability social care settings. People with intellectual disability and challenging behaviour have already been involved centrally with the design, development and pilot evaluation of WCW and will also be fully involved throughout this trial.

**Trial registration:**

Registered on the International Standard Randomised Controlled Trial Number registry on 8th December 2015: ISRCTN53763600.

**Electronic supplementary material:**

The online version of this article (doi:10.1186/s13063-017-2175-1) contains supplementary material, which is available to authorized users.

## Background

Individuals with intellectual disability (ID) often engage in behaviours that are labelled as ‘challenging’ (challenging behaviours; CB). CBs are actions that may place the individual at risk of harm or exclusion, or may place other people (e.g. carers) at risk of harm [1]. Thus, CBs are defined socially, in terms of their impact. Behaviours labelled as challenging typically include actions such as anger and aggression, self-injurious behaviours (e.g. self-biting, hitting body parts against objects, scratching and gouging), destruction of property, and inappropriate or risky social behaviour. CBs are, by definition, a significant challenge for services and impact negatively on the quality of life of people with ID. CBs are also related to risk of abusive practices (cf. Winterbourne View scandal exposed by BBC Panorama), increased carer stress [2, 3], and high cost of support services [4]. High quality epidemiological research suggests that 18–19% of adults with ID known to services engage in CB that has a significant impact on their lives [5, 6].

A number of contextual factors represent an increased risk for the emergence and ongoing maintenance of CB, including genetic factors, other biological vulnerabilities and the severity of ID and communication impairments [7]. However, the underlying theory is clear in that the behaviour of other people (especially support staff, family carers) in the environment of people with ID is the most significant factor [7]. Other people create the conditions in which CBs become an effective communication tool (e.g. by placing inappropriately difficult demands on people with ID). In addition, other people respond to CB in ways that ensures its longer term maintenance by ‘reinforcing’ the behaviour (e.g. removing demands when CB occurs). These patterns of support staff behaviour are targeted for change in the dominant treatment approach, termed positive behaviour support (PBS), used in ID services. PBS is recommended for use in UK ID services (e.g. Royal College of Physicians, British Psychological Society, & Royal College of Speech and Language Therapists ‘Unified Approach’ 2007 report [8]).

Although strongly informed by person-centred values, a limitation of PBS is that it does not include explicit elements to either increase support staff motivation to engage in changing their own behaviour or to engender attitude change. The latter is significant, since support staff beliefs and attitudes are a core part of theoretical models of why staff behave in ways that increase the risk for the development and maintenance of CB [7, 9, 10]. In addition, even though behaviour support plans (the core of PBS intervention) are developed for many individuals with CB, these plans are often not implemented by staff [11]. Therefore, a training intervention for staff designed to change attitudes towards people with ID and CB is needed. Our review of the support staff training literature in ID settings identified no existing evidence-based training course to increase empathy and to change support staff attitudes towards those with CB [12]. Similarly, MacDonald and McGill’s [13] systematic review of PBS staff training included no studies with this focus and no outcome measures designed to assess staff empathy.

We developed the Who’s Challenging Who (WCW) training course for support staff to address this identified need for training. The WCW training course was informed by five domains of evidence during its development:

1. User involvement work clarified that support staff needed an increased empathy for people with CB (as this would likely reduce the risk for many CB incidents), and that user stories and other perspectives could be used to affect change.
2. WCW’s theory of change focused on theories of attitude change, especially the Contact Hypothesis [14]. Thus, to change support staff attitudes, they needed to come into direct contact with people with CB, in a shared endeavour, where the people with CB were in a valued social role.
3. Discussion with ID service providers made it clear that any additional training that would either stand alone or be included in existing PBS treatment approaches must be short (no more than half a day in length).
4. We carried out a systematic review and qualitative synthesis of research on the direct experience of people with ID’s of being labelled as ‘challenging’, and being in receipt of CB services and physical restraint [15].
5. We carried out a second systematic review and qualitative synthesis of the experiences of carers of people with ID and CB, with a particular focus on carers’ experiences of and views about services [16].

In a pilot study [12], we trained two adults with ID and CB using the Co-Trainer Training manual and then these trainers co-delivered 10 small group training courses to 76 ID services staff with a trainer without disability (using the WCW manual). We found positive changes in staff-reported empathy towards people with ID and CB, increased confidence in dealing with CB, and positive changes in staff attitudes (specifically, an increased tendency to perceive people with ID as like themselves, and more positive attitudes towards the empowerment of people with ID) [12]. The pilot study demonstrated that we could recruit staff from services to attend training, manualised delivery by two trainers (one with ID) was feasible, and that there was some indication of the intended positive change in outcomes for support staff. The effectiveness of the WCW training course now needs to be evaluated in a large scale robust research trial.

## Methods

### Primary objective

The primary objective is to assess the effectiveness of the WCW staff training course to increase empathy of social care staff working in residential homes for people with ID who may display CBs, compared to a waiting list control group.

### Secondary objectives

The secondary objectives are to evaluate whether WCW, compared to a waiting list control, impacts ID social care staff with regards to (1) self-efficacy in working with people with CB, (2) attitudes towards people with ID and CB, and (3) work-related well-being.

In relation to service users with ID, to explore and evaluate whether WCW impacts (1) recorded incidents of aggressive CB within residential settings and (2) recorded use of restrictive interventions for CB within residential settings.

A qualitative component of the study will explore a number of perspectives designed to inform future uptake of WCW into social care practice, including (1) staff experience of receiving the training and (2) barriers and facilitators for the WCW Action Plans in social care practice.

### Study design

The trial will be a cluster randomised controlled trial. Overall, 118 residential settings will be recruited into the trial and randomised to the either the WCW training arm or to a waiting list control group. At each residential setting, two members of staff will be recruited; one manager/lead staff member along with one other support staff member. In both arms of the trial, those staff taking part will continue to receive training as usual (on ID social care and specifically on CB if provided) following their organisations’ ongoing staff development policies. Recruitment will be in two phases with approximately 58–60 residential settings per phase. Phase 2 of the trial will immediately follow the 20 week data collection time point for phase 1. Randomisation will occur at one point in time for each phase and will be carried out by a study-independent statistician from the Centre for Trials Research using a dynamic balancing algorithm specifically designed for cluster randomised trials [17]. Allocations will be stratified by residential setting size, geographical region and phase of recruitment (i.e. phase 1 or 2) in a ratio of 1:1. A Standard Protocol Items: Recommendations for Interventional Trials (SPIRIT) checklist is provided in Additional file 1.

### Eligibility criteria

Residential settings and staff must fulfil all of the inclusion criteria and none of the exclusion criteria as detailed in Table 1. For homes found to be ineligible at screening, basic demographic data regarding reasons for non-eligibility will be recorded on screening logs.

**Table 1:** Inclusion and exclusion criteria

Inclusion criteria for residential settings:	Exclusion criteria for residential settings:
Provides services via English/Welsh publically funded contracts (Local Authorities, Clinical Commissioning Groups)	‘Inpatient’ hospital facility (typically NHS or independent providers)
Provides support to between one and 10 people with intellectual disability (ID)	The manager does not receive approval from the service provider organisation for the residential settings to be a part of the study
Provide at least some 24-h support for people with ID	
Is in a community location	
Provides care for at least one person with ID who engages in aggressive challenging behaviour	
Manager (or equivalent lead staff member) and one other support staff member can be released to attend a ‘Who’s Challenging Who?’ training session together	
Inclusion criteria for staff:	Exclusion criteria for staff:
One staff member will be the manager or other lead staff member (as defined by the service provider organisation)	Staff do not provide their consent to take part in the research
The second staff member will be a direct support worker whose role is no more than 50% in administrative/staff management tasks	Inadequate English reading skills prevent completion of the questionnaire measures
Both staff work at least a 0.70 full time equivalent or more	

### Care home and participant selection

Residential settings will initially be recruited from the West Midlands area using publically available data from the Care Quality Commission. If recruitment is slow, we will extend the geographical spread throughout England and Wales to meet the recruitment target. The recruitment strategy will also include direct contact with national (England and Wales) ID social care services provider organisations with a view to exploring their interest in the research. In negotiation with some providers, it may be necessary to extend recruitment of residential settings nationwide.

### Recruitment process

Residential setting managers will be contacted for a structured telephone screening interview based on the inclusion and exclusion criteria as well as additional information (e.g. total number of users in the residential setting, size of staff group, postal code of the residence). To establish whether at least one user engages in aggressive CB at least weekly on average, items from the aggression/destruction scale of the Short Form of the Behavior Problems Inventory [18] will be used as prompts.

### Outcome measures

**Fig. 1:**
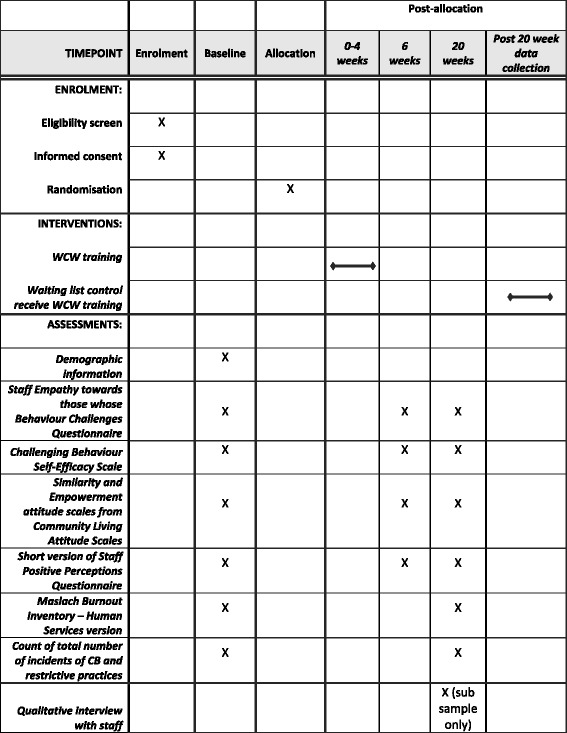
Standard Protocol Items: Recommendations for Interventional Trials (SPIRIT) figure – Schedule of enrolment, interventions and assessments

These will be collected prior to randomisation (at baseline) and then from both arms at 6 weeks post-randomisation (following WCW training), and again at 20 weeks post-randomisation (Fig. 1).

#### Eligibility screening measures

Items from the aggression/destruction scale of the Short Form of the Behavior Problems Inventory [18, 19].

#### Primary outcome measures

Staff Empathy towards those whose Behaviour Challenges Questionnaire (SECBQ) [12] (5 items: 1 minute).

#### Secondary outcome measures

Challenging Behaviour Self-Efficacy Scale [20] (5 items: 1 minute); Similarity and Empowerment attitude scales from the Community Living Attitude Scales [21] (25 items: 5 minutes); Maslach Burnout Inventory – Human Services version [22] (22 items: 5 minutes); Short version [23] of the Staff Positive Perceptions Questionnaire [24] (11 items, 2–3 minutes); and recorded incidents of challenging behaviour and use of restrictive practices in each residential setting.

Measures for staff self-report will be administered in the form of a questionnaire. Additional measures focused on CB incidents and use of restrictive practices at the residential setting level will be included for residential setting managers to complete. To capture these data, we will use definitions developed to capture the same outcomes in earlier research on aggressive behaviour in ID settings [25, 26]. Although incident data are recorded using varied systems, our methodology captures data according to decision rules that can be used with any system typically in use. A similar data extraction and coding system will be used to count use of restraint procedures recorded in the residential settings across all users. In both methods, incidents over the 16 week period preceding data collection points will be included. In addition to the measures listed, a short staff demographic details questionnaire (e.g. age, sex, qualifications, work role) will be included for staff at baseline.

### Intervention – WCW training programme

**Fig. 2:**
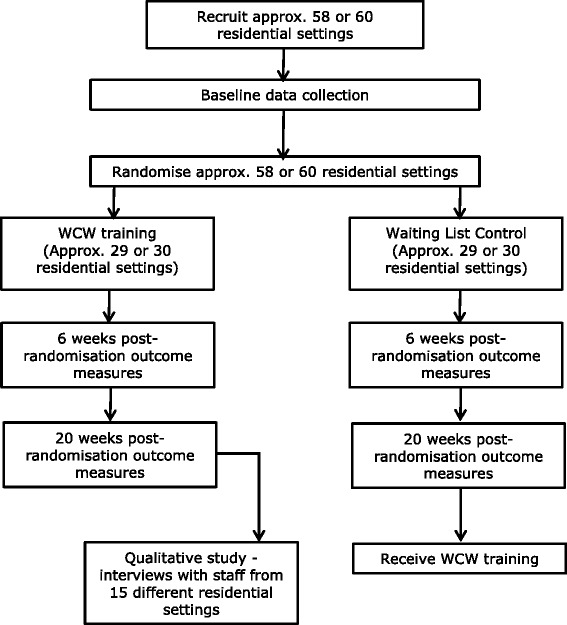
Summary flow chart (Note: Process repeated to recruit a total of 118 residential settings)

Following baseline, those allocated to receive the intervention will attend a training session (anticipated up to 12 staff per session). Those allocated to the waiting list control will be informed that their training session will available to them after the 20 week follow-up data collection time point (Fig. 2).

The training is a half-day session delivered by a trainer with ID and CB supported by a trainer without ID. WCW was developed jointly with people with ID and CB and the training course is fully manualised. Trainers with ID and CB are trained to deliver WCW using a process that is also manualised (Co-Trainer Training). WCW training covers the following topics (from the perspective of people with ID and CB):

- Communication and how staff listening can prevent escalation of CB
- How the living environment contributes to frustration and CB
- The experience of being physically restrained
- What it is like to be on medication ‘for’ CB
- Experiences of feeling excluded because of CB
- Unhelpful attitudes and behaviour of support staff, and a discussion of positive qualities that contribute to good support/care

At the end of the WCW training session, attendees write an Action Plan of what they will do differently in their service on return. These plans are shared with the group for feedback from the trainers and other attendees. Following WCW training, the trainer without ID will contact each residential setting manager/lead for a 30 min coaching session (telephone, Skype or similar voice connection system). The trainer will follow a standard protocol for this coaching session to finalise the Action Plan and how this will be introduced to, adjusted and then agreed with the staff team (e.g. as a part of a regular team meeting). After the Action Plan has been agreed with the team, a further 30 min coaching session will focus on the implementation and monitoring of the Action Plan. WCW training ends with this second coaching session.

In summary, WCW training includes four core components, namely (1) staff from a residential setting attend a half day training course; (2) these staff develop an initial Action Plan for their setting that is shared with the group receiving the training and the trainers; (3) the trained staff then receive an individualised short coaching session focused on how to disseminate the messages from the training and agree an Action Plan with the whole staff team of the residential setting; and (4) a follow-up coaching session on how to implement the agreed plan and to monitor its implementation (including the need for ongoing review with the staff team). Thus, WCW is not just a half-day training course.

The rationale for the additional short coaching elements is to ensure that learning from WCW training is translated into practical actions within each setting. A limitation of much existing staff training is the lack of planning to generalise the learning gains into everyday social care practice. A recent meta-analysis of factors affecting outcomes in staff training in ID settings suggested that a combination of content training with coaching is more effective than classroom/workshop training alone [27]. Other data on training in ID contexts has highlighted the importance of supportive managers [28], hence our focus on training the residential setting manager or other senior staff member alongside other staff in each setting.

### Adherence

Following the 20-week post-randomisation data collection, the research assistant (RA) will code the first eight WCW training sessions (audio-recorded) for fidelity of delivery according to the manual. This will be done using a checklist developed from the manual. Each WCW training element will be rated 0–2 (0 = missing, 1 = partially delivered, 2 = fully delivered according to the manual). All eight training sessions will be coded for inter-rater agreement by the chief investigator. Once inter-rater reliability has been established, the RA will go on to code fidelity for the remaining eight training sessions.

### Qualitative interviews and process evaluation

After the first phase of the study, the RA will recruit managers and other trained staff members from 12 to 15 residential settings (one interviewee per setting) to participate in a semi-structured interview. The interview will focus on any changes in the residential setting that the staff have noted, their experience of any changes in staff attitudes and behaviours, their relationships with and treatment of individuals with and without CB, and perspectives on the process of developing the Action Plan and both facilitating factors and barriers to implementation of these plans. Staff also complete a post-training evaluation questionnaire before leaving the session. All of the WCW trainers will also be interviewed, primarily about their experience of being a trainer, delivering the training, and their experience of being trained up for the role.

### Data on costs of the WCW training

WCW training is not hypothesised to result in cost savings due to a change in service use by people with ID in the residential settings. However, we will directly estimate the cost of delivering WCW training (including initial recruitment of co-trainers with ID, their training, support, venues, etc.). At the end of the study, the RA will carry out an additional structured telephone interview with five or six residential setting managers (chosen purposively to represent the range of sizes and types of residential setting recruited) with a view to estimating the costs of sending staff members to the WCW training sessions (replacement staff time, travel, and any other costs).

### Safety reporting

No adverse events are expected; however, they will be collected, recorded and reported in accordance with good clinical practice and the requirements of the research ethics committee. An adverse event (AE) is any untoward medical occurrence in a trial participant which does not necessarily have a causal relationship with this treatment. An AE can therefore be any unfavourable and unintended sign (including abnormal laboratory finding), symptom or disease. A serious AE is any AE that results in death, is life-threatening, requires hospitalisation or prolongation of existing hospitalisation, results in persistent or significant disability or incapacity, consists of a congenital anomaly or birth defect, or other medically important condition. The Chief Investigator may carry out urgent safety measures to protect participants from immediate harm.

### Trial management and oversight

A Trial Steering Group consisting of an independent Chair and three other independent members (one manager of a social care service for people with ID and CB, one statistician, and one other expert in ID research or practice) will provide trial oversight and will act as Data Monitoring Committee. One of the WCW co-trainers will also be a full member of the Trial Steering Group. An Advisory Group will also be convened to support the trial. It will have an independent Chair, a co-trainer and two additional ID experts. Both groups will meet three times over the course of the trial.

### Sample size determination

The sample size calculation is based on the results from Hutchinson et al. [12], where an effect size of 0.50 (standardised mean difference) for the staff empathy score (primary outcome) was observed. With alpha set at 0.05 and power at 90%, the unadjusted sample size required is 172 (86 per arm) staff. With cluster size as two staff per residential settings and allowing an intracluster correlation of 0.10, the variance inflation factor is 1.1. Therefore, a total of 189 staff are needed (95 per trial arm). Building in a 20% loss to follow-up means an estimated 237 staff are required. This calculation is the basis for the sample size for this study of 118 residential settings.

### Main analysis

Statistical analysis will be conducted based on the intention-to-treat principle. A two-level (staff nested within residential settings) analysis of covariance (ANCOVA) will be fitted to provide a between-group comparison of mean SECBQ scores at 20-weeks post-randomisation follow-up, adjusted for baseline SECBQ scores. The analysis will also adjust for residential setting size and region (stratification factors at randomisation).

Secondary outcomes measured at the staff level (self-efficacy, similarity and empowerment attitudes, work stress and positive experiences) will be analysed as above; two-level ANCOVA will be fitted to measures at follow-up, adjusted for the corresponding baseline covariates, region and residential setting size. Secondary outcomes measured at the residential setting level (incidents of aggressive CB and incidents of recorded restraint procedures) will be analysed using an appropriate single level regression analysis that adjusts for baseline levels of the outcome, region and residential setting size.

All main statistical analysis will be conducted on complete cases (i.e. all randomised residential settings and staff providing follow-up data). However, to investigate the impact of missing outcome data on the conclusions drawn from the trial, missing mechanisms will be explored and appropriate imputation methods applied via sensitivity analyses. Baseline characteristics will be presented by trial arm. The WCW training fidelity data will be summarised descriptively for inclusion in the main results paper.

### Exploratory analysis

Two exploratory mediation analyses will be conducted to investigate whether any effect of the intervention on incidents of aggressive CB and incidents of recorded restraint procedures are mediated through effects on staff empathy. While the study is not powered to detect small differences in subgroups, we plan to conduct exploratory subgroup analyses of any differential treatment effects on the primary outcome by factors such as length of time staff have worked in social care, proportion of people with CB in each residential setting, and the length of time staff have been employed in the specific residential setting.

### Analysis of interview data

The data from interviews will be analysed using thematic analysis. The qualitative study will be reported separately and will focus on the experience of staff participating in WCW training and emerging recommendations about the process of larger scale implementation of WCW in social care. The trainers will also collate feedback on WCW training courses (from end-of-session training feedback questionnaires used as a part of the WCW course) and these data will also be analysed descriptively and used to inform the analysis resulting from the interviews with staff.

## Discussion

This trial will assess the effectiveness of the WCW training course for staff in residential settings to increase their empathy towards people with ID and CBs compared with a waiting list control group. Changing factors that either contribute to the development or maintenance of CB or affect the quality of support and services delivered to people with ID and CB, and their families/carers, has considerable potential to improve social care practice and outcomes. Although a part of the national response to the Winterbourne View care scandal was the recognition of the role of carer and user involvement in services, there is a lack of evidence-based approaches to making that user voice heard and for it to affect social care practice on a day-to-day basis. As well as being grounded in longer-term research and theoretical development, WCW offers a practical solution to the inclusion of the perspectives of users with ID and CB in staff training to directly impact social care practice.

## Trial status

The trial is sponsored by the University of Warwick and is currently on-going and open to follow-up. This manuscript has been drafted according to version 1.3 (26th April 2016) of the trial protocol. The protocol has been written according to the SPIRIT statement. The final report will follow the CONSORT statement.

## Additional file

Additional file 1: Standard Protocol Items: Recommendations for Interventional Trials (SPIRIT) Checklist. (DOC 227 kb)

## Abbreviations

AE: Adverse event
ANCOVA: Analysis of covariance
CB: Challenging behaviour
ID: Intellectual disability
RA: Research assistant
SECBQ: Staff Empathy towards those whose Behaviour Challenges Questionnaire
WCW: Who’s Challenging Who
